# Anatomical and symptomatic mid-term outcomes of patients with isolated anterior compartment defect repair or stress urinary incontinence: Anatomical and symptomatic outcomes of anterior compartment repair or SUI

**DOI:** 10.1186/s12905-023-02556-0

**Published:** 2023-08-23

**Authors:** İsmail Gökbel, Mehmet Ferdi Kinci, Deniz Akin Gökbel, Ahmet Akın Sivaslioğlu

**Affiliations:** 1 Obstetrics and Gynecology Department, Menteşe State Hospital, Muğla, Turkey; 2 Obstetrics and Gynecology Department, Muğla Sıtkı Koçman University Education and Research Hospital, Muğla, Turkey

**Keywords:** Pelvic organ prolapse (POP), Lower urinary tract symptoms (LUTS), Stress urinary incontinence (SUI), Anterior compartment defect (ACD)

## Abstract

**Background:**

An evaluation of preoperative and postoperative 12th month Pelvic Organ Prolapse Quantification (POP-Q) and Lower Urinary Tract Symptoms (LUTS) changes in patients operated for the diagnosis of isolated anterior compartment defect (ACD) or Stress Urinary Incontinence (SUI).

**Method:**

Patients who were diagnosed with isolated ACD or SUI were retrospectively analyzed at urogynecology unit of our tertiary referral center. All pelvic examinations were performed by the same experienced urogynecologist. Pre-operative and post-operative 12th month POP-Q scores and the responses to a detailed LUTS questionnaire in the unit were assessed.

**Results:**

Of the 90 patients with isolated ACD or SUI, midurethral sling with mini-sling and retropubic transobturator tape methods was applied in 24, iliococcygeal fixation in 28, trapezoid repair in 9 patients, anterior bridge operation in 14, and plication of pubocervicovaginal fascia to the cervical ring in 15. We compared the POP-Q score and pre and post-operative 12th month LUTS. Between pre and post-operative 12th month, there was a statistically significant difference at Aa and Ba points (p < **0.00, 0.001**). Comparative LUTS questionnaire showed statistically significant differences in stress urinary incontinence, frequency, urgency, abnormal emptying, nocturia, pelvic pain (p: **<0.001, p < 0.001, p: <0.001, p:0.001, p:<0.001, p:0.003**, respectively).

**Conclusion:**

Anatomical and symptomatic recovery is achieved with appropriate surgical intervention in women with isolated ACD or SUI. When LUTS were evaluated in terms of symptomatic recovery, they were found to be related not only to symptoms involving the anterior compartment, but also to symptoms involving other compartments.

## Background

Pelvic organ prolapse (POP) is seen in 40–60% of multiparitous women [1]. The anterior compartment is the most frequently affected and the most difficult part to repair [1]. Anatomically, the anterior compartment is the site between the external urethral meatus and the anterior lip of the cervix (vaginal cuff in hysterectomized women) and is supported below by the pubocervicovaginal fascia (PCVF). Depending on the deterioration of the anatomical relations here, central cystocele, cystocele with paravaginal defect, high cystocele and/or urethrocele occur. These can be isolated or combined [2].

Integral theory (IT) considers the pelvic floor as a whole [3]. Any anatomical defect in the pelvic floor may be associated with any or more of a wide spectrum of lower urinary tract symptoms (LUTS). About half of women have LUTS [4]. However, interestingly, there is no linear correlation between POP stage and LUTS severity [5]. Women with ACD most frequently have a bulge, lump or protrusion coming down from the vagina, a pelvic heaviness, a dragging sensation in the vagina, stress urinary incontinence (SUI), and fecal incontinence, frequency and urgency [6, 7].

In the choice of treatment, an evaluation should be made with a detailed history (including questionnaires) and physical examination [Pelvic Organ Prolapse Quantification (POP-Q) and specific tests such as Q tip test, Urodynamics studies], and the defect should be repaired and treated using natural tissue (colporrhaphy anterior, iliococcygeal fixation, etc.) or, rarely, synthetic materials [8, 9].

In the current study, we analysed the anatomical and symptomatic changesthe patients with isolated ACD in pre and post-operative 12th month.

## Methods

This was a retrospective study conducted between January 2018 and January 2021 at the urogynecology units of Mugla University Education and Research Hospital. The study was initiated after obtaining approval from the ethical review board (Date:23.06.2021, Number:13/III). The patients who were admitted to the urogynecology unit with any kind of prolapse were analyzed retrospectively. Informed consent wasn’t obtained because the study was planned retrospectively. All the information was retrieved from the urogynecologic medical record forms. The patients’ demographic data and past medical and surgical histories (age, menopausal status, obstetric history, body mass index (BMI), coexisting comorbidities and tobacco use) were collected. They were included in the analysis if they had isolated ACD with no clinically significant apical and posterior compartment defects or the patient with Stress Urinary Incontinence (SUI). Women with a urogynecologic operation history were excluded from the analysis.

The baseline clinic visit included a standard clinical examination and a detailed urogynecologic examination including evaluation of all three vaginal compartments (anterior, apical, and posterior). All pelvic examinations and surgeries were performed by the same experienced urogynecologist (Prof. Dr. AAS). The POP-Q staging system was used to assess prolapse [10]. The responses to a LUTS questionnaire that is identified by Peter Petros in IT [3], were also assessed. Also, this is a part of routine urogynecologic evaluations in our unit. The LUTS were noted as follows: SUI, frequency, urgency, constipation, abnormal emptying, nocturia, fecal incontinence, pelvic pain, coital incontinence, and vaginal winding were also noted. Patients were evaluated as preoperative and postoperative 12th month andthen compared with each other.

### Statistical analysis

None of the continuous data had a normal distribution. Therefore, he used the non-parametric Wilcoxon test in all of them. The median value was calculated because age, gravity and BMI data did not fit the normal distribution. Also, mean ± SD values have been added. McNemar test was used because there were dependent groups in categorical data.

## Results

**Fig. 1:**
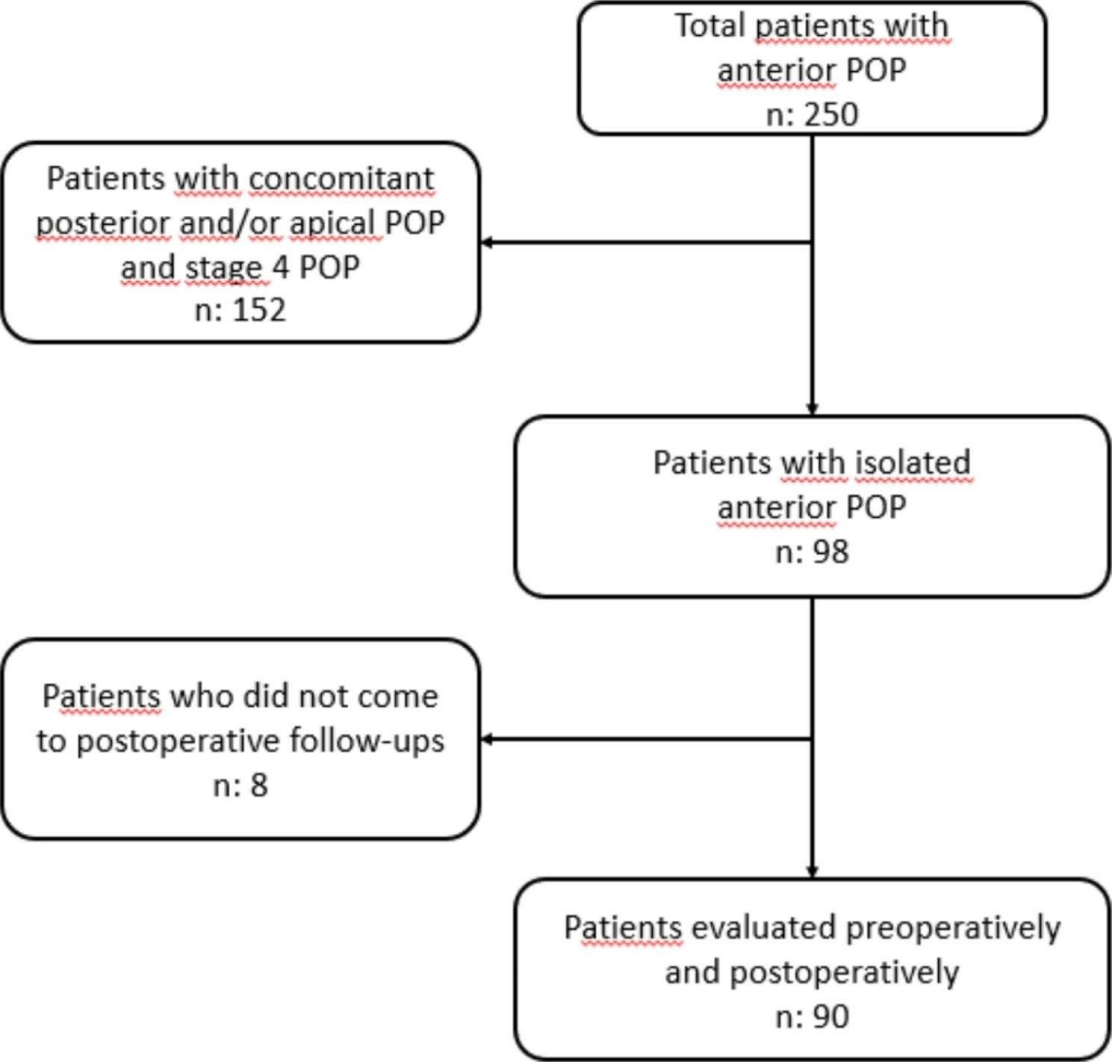
Selection of the study populations

A total of 250 patients with every kind of prolapse who had not undergone urogynecologic surgery before were evaluated within the scope of the study. A total of 152 patients with posterior and/or apical compartment defects were excluded from the study. The remaining 98 patients were included in the study program after surgery. Eight patients were excluded from the study because they did not come for post-operative follow-ups (Fig. 1).

The mean age of the patients was 61.72 ± 8.47 years. Of the patients, 12 (%13.33) were pre-menopausal, while 78 (%86.66) were post-menopausal. The parity of the patients was 2.76 ± 0.78. Only 8 (8.88%) of the patients were nulliparous. The BMI of the patients was 27.40 ± 4.12 kg/m^2^. A total of 40 (44.44%) patients were obese with a BMI ≥ 30 kg/m^2^. The demographic data of the patients participating in the study are given in Table 1. When co-morbid diseases were evaluated, hypertension was found in 38 (42.2%) patients, diabetes mellitus was found in 30 (33.33%) patients, and hyperlipidemia was found in 22 (24.44%) patients. Twenty-one (23.33%) of the patients were smokers.

**Table 1:** Demographic Characteristics of the patients

	Mean ± SD	Median (Range)
Age (year)	61.72 ± 8.47	63.00 (41.00, 79.00)
Parity (n)	2.76 ± 0.78	3.00 (0.00, 5.00)
BMI (kg/m^2^)	27.40 ± 4.12	28.10 (19.25, 38.40)

Of the remaining 90 patients with isolated ACD, midurethral sling with mini-sling and retropubic transobturator tape methods was applied in 24, iliococcygeal fixation in 28, trapezoid repair in 9 patients, anterior bridge operation in 14, and plication of PCVF to the cervical ring in 15 [11–13]. Pre-operative and post-operative 12th month POP-Q and LUTS were compared.

Pre-operative and post-operative evaluations were performed on the patients according to their POP-Q classification. While the Aa point was 0.87 ± 1.19 pre-operatively, it was − 1.72 ± 0.87 post-operatively (p < 0.00). Ba point was 2.21 ± 1.49 pre-operatively and − 1.74 ± 1.10 post-operatively (p < 0.001). There was no statistically significant change in C, D, Ap and Bp in which the apical and posterior compartments could be evaluated (p > 0.05). Pre-operative and post-operative POP-Q values of the patients are given in Table 2.

**Table 2:** Pre-operative and Post-operative POP-Q values

	Pre-operative (mean ± SD)	Post-operative (mean ± SD)	P
Aa	0.878 ± 1.195	-1.728 ± 0.878	< 0.00*
Ba	2.217 ± 1.490	-1.744 ± 1.107	< 0.001*
C	-3.478 ± 1.440	-3.656 ± 1.367	0.432
D	-5.384 ± 1.705	-5.479 ± 1.620	0.756
Ap	-2.278 ± 0.924	-2.378 ± 0.712	0.713
Bp	-2.311 ± 0.932	-2.422 ± 0.687	0.710
TVL	7.900 (0.849)	7.900 (0.849)	1
Gh	4.557 (0.969)	4.511 (0.910)	0.833
Pb	2.119 (0.429)	2.102 (0.409)	0.720

*: p < 0.05, statistically significant.

**Fig. 2:**
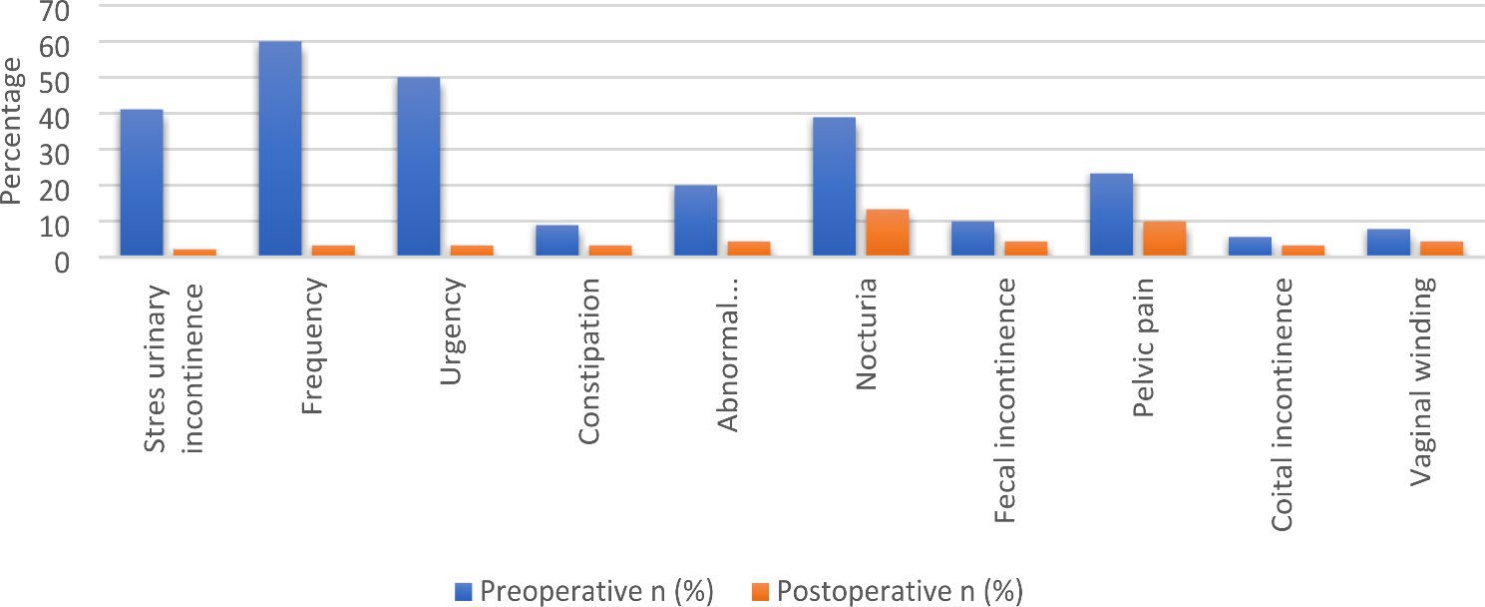
Comparison of pre-operative and post-operative LUTS

**Fig. 3:**
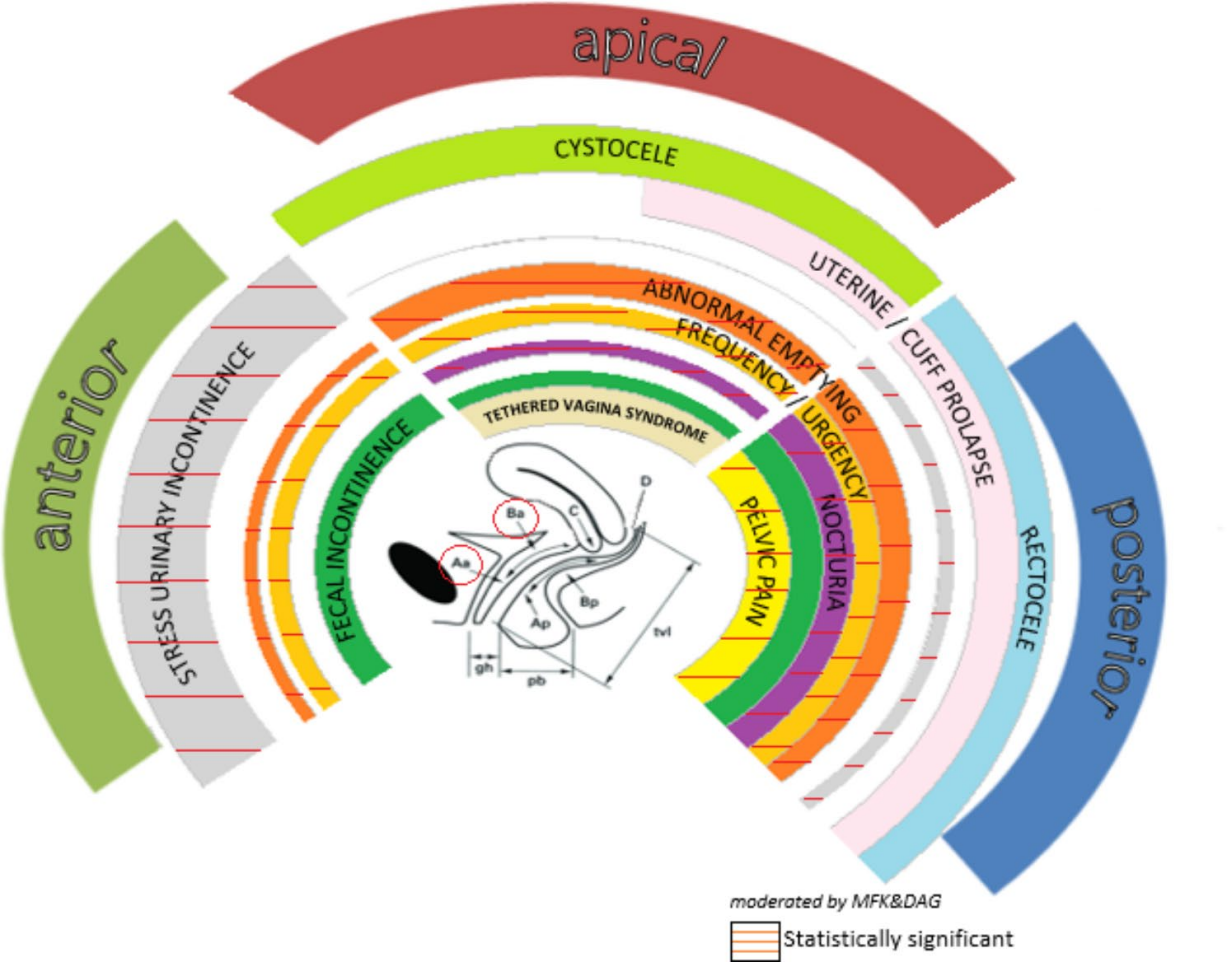
Comparison of pre-operative and post-operative LUTS (modified by MFK&DAG from Peter Petros “The symptom-based Pictorial Diagnostic Algorithm of IT”)

In the pre-operative and post-operative comparison of LUTS, statistically significant improvement was observed in terms of SUI, frequency, urgency, abnormal emptying, nocturia and pelvic pain (p: <0.001, p < 0.001, p: <0.001, p:0.001, p:<0.001, p:0.003). There was no statistically significant difference in terms of constipation, fecal incontinence, coital incontinence, and vaginal winding (p > 0.05). The pre-operative and post-operative LUTS values of the patients are given in Table 3, (Figs. 2 and 3). There were no women with de-novo SUI.

**Table 3:** Pre-operative and 12th month Post-operative LUTS evaluation

	Pre-operative n (%)	Post-operative n (%)	P
Stress urinary incontinence	37 (41.1%)	2 (2.2%)	< 0.001*
Frequency	54 (60.0%)	3 (3.3%)	< 0.001*
Urgency	45 (50.0%)	3 (3.3%)	< 0.001*
Constipation	8 (8.9%)	3 (3.3%)	0.182
Abnormal emptying	18 (20.0%)	4 (4.4%)	0.001*
Nocturia	35 (38.9%)	12 (13.3%)	< 0.001*
Fecal incontinence	9 (10.0%)	4 (4.4%)	0.182
Pelvic pain	21 (23.3%)	9 (10.0%)	0.003*
Coital incontinence	5 (5.6%)	3 (3.3%)	0.723
Vaginal winding	7 (7.8%)	4 (4.4%)	0.546

*: p < 0.05, statistically significant.

## Discussion

The current study investigates the effects of surgical correction of isolated ACD and SUI on POP-Q scoring system and LUTS. According to the POP-Q scoring system, anatomical improvement was detected in the Aa and Ba points. In the LUTS evaluation, improvements were seen in SUI, frequency, urgency, abnormal emptying, nocturia and pelvic pain.

ACD is the most common type of prolapse. Therefore, ACD repair is the most commonly performed POP surgery, either primary or combined [14]. Although the probability of recurrence varies according to factors such as age, parity, increased abdominal pressure, and tissue weakness, the long-term success rate varies between 30 and 70% [15]. No universal surgical technique has been defined to improve the high risk of long-term failure [7, 16]. Therefore, treatment should be individualized. The evaluation of most of the studies in the literature was prepared as a single surgical procedure or as a comparison of two surgical procedures [9, 11, 14]. In this study, patients underwent midurethral sling, iliocoxygeal fixation, trapezoid repair, anterior bridging, and plication of PCVF to the cervical ring. Different operational techniques have been used because we tried to cure the underlying pathology and, hence, to treat the ACD. As an example, we used bridge technique for central cystoceles however the traphezoid restoration was carried out in patients who had lateral defect. Similarly the patients who had lateral and ACD were treated with iliococygeal fixation. The operations were varied because the defects in anterior compartment were varied. Regarding the quantity of operations; we were not aiming to compare the effectiveness between operations; therefore, we believe the number of each operation is not important. The preoperative Aa and Ba values werre significantly different from postoperative Aa and Ba values (p < 0.00 and p < 0.001, respectively). This shows us that although variable techniques used in accordance with the underlying pathology; the anterior compartment had been anatomically repaired. And now, we are able to evaluate the effects of surgical correction in patients concerning LUTS.According to the symptom-based Pictorial Diagnostic Algorithm of IT [3], one can predict the defect zones by the symptoms query, SUI, fecal incontinence, frequency and urgency are common symptoms of ACD. In the current study, when operative symptoms were evaluated, SUI, frequency, urgency, and nocturia were the most common symptoms. The results obtained are in line with IT in this respect.

In the study by Kılıç et al., pre-operative and post-operative 12th month POP-Q evaluation was performed in 30 patients who underwent “bilateral iliococcygeal fixation of the pubocervical fascia” operation [8]. Accordingly, statistically significant improvement was observed in Aa, Ba, Ap, Bp, C and D points in the POP-Q evaluation. In the study by Sivaslıoğlu et al., site-specific surgery was applied to 45 patients with cystocele and mesh surgery was performed to 45 patients [17]. An improvement was observed in the Aa, Ba, C, and Gh points in the mesh-applied patient group, and in the Ba and Gh points in the site-specific group. In the study by Başer et al., site-specific surgery, transvaginal mesh placement and anterior colporrhaphy were performed in 30 patients with isolated ACD [11]. As a result of the present study, a statistically significant improvement was observed in Aa, Ba in the POP-Q evaluation at the post-operative 7th month, similar to Kılıç et al.‘s study [8]. In the POP-Q evaluation, it is considered that the results obtained depend on the surgical procedures performed against ACD. The lack of change in the evaluation of the apical and posterior compartments is attributed to the fact that the cases consisted of cases with isolated ACD.

In the study by Kılıç et al., pre-operative and post-operative 12th month LUTS evaluation was performed. In the evaluation of LUTS, a statistically significant improvement was observed in urgency, frequency, nocturia, hesitancy, interrupted stream, abnormal emptying values, and there was no significant difference in terms of UI [8]. In the study by Sivaslıoğlu et al., site-specific surgery was applied to 45 patients with cystocele and mesh surgery was applied to 45 patients [17]. According to the results of this study, improvements were seen in abnormal emptying, frequency, in the site-specific surgery group, and in abnormal emptying, frequency, urgency, and pelvic pain in the mesh surgery group. In Başer et al.‘s LUTS evaluation, there was improvement in all LUTS, including urgency, urge incontinence, frequency, abnormal emptying, hesitancy, nocturia, dysuria, nocturia, pad used and pelvic pain, respectively, pre-operatively and post-operatively [11]. In the current study, improvement was found in SUI, frequency, urgency, abnormal emptying, nocturia and pelvic pain. Although the study was on surgical interventions in patients with isolated ACD, symptoms that were more related to the apical and posterior compartments were also improved compared to IT. This is attributed to the fact that the pelvic floor is a whole and anatomical correction to be made in a single compartment can positively affect other compartments.

### Limitations

In this study, post-operative 12th month results were evaluated, and these results can be interpreted as medium-term results. We plan to publish long-term results with future studies. Another limitation of the study were we didn’t use any quality of life questionnaire. Also, the number of patients is too low to allow a comparison of the different operation techniques used during this study. We are planning to conduct studies with the same techniques with higher number of patients.

## Conclusion

Anatomical and symptomatic recovery is achieved with appropriate surgical intervention in women with isolated ACD. Anatomical recovery is effective on the Aa and Ba points of the anterior compartment. Symptomatic recovery, on the other hand, positively affects not only the anterior compartment, but also the symptoms related to other compartments. Therefore, surgical treatment of cases with isolated ACD should be individualized.

## Data Availability

The datasets analysed in the study are available from the corresponding author on reasonable request via.

## List of Abbrevations

ACD: Anterior Compartment Defect
LUTS: Lower Urinary Tract Symptoms
POP: Pelvic Organ Prolapse
PCVF: Pubocervicovaginal Fascia
IT: Integral Theory
SUI: Stress Urinary Incontinence
BMI: Body Mass Index
POP-Q: Pelvic Organ Prolapse Quantification
